# The contribution of family medicine to the health system in Somaliland

**DOI:** 10.4102/phcfm.v13i1.3051

**Published:** 2021-09-06

**Authors:** Abdikadir O. Rabiile, Mohamed A. Abdillahi, Mohamoud H. Abdi, Rahma I. Yasin, Mubarik A. Magan, Tim Fader

**Affiliations:** 1 Department of Family Medicine, Amoud University, Borama, Somalia

**Keywords:** family medicine, family medicine training, employment, emigration, health system, Essential Package of Health Services, primary health care

## Abstract

Somaliland’s first specialty training programme for physicians was a master’s degree in Family Medicine that began at Amoud University in 2012. A survey of the 24 Family Medicine graduates working in Somaliland demonstrates their clinical and leadership impact on the health system and their contribution to higher education. The specialists directly contribute to the health and education priorities of the government of Somaliland.

## Introduction

In a scoping review of the state of family medicine in sub-Saharan Africa (SSA), Flinkenvlogel et al. sought answers to five key questions.^1^ The fifth question asked, ‘Where are Family Physicians deployed in SSA health systems?’ In 2012 Amoud University started the first Family Medicine training programme in Somaliland. This short report focuses on the initial contribution of family medicine to the health system by studying the employment of the first 29 graduates of the programme.

Somaliland is a self-declared independent country in the Horn of Africa. It was formerly the British Somaliland Protectorate, united with Italian Somaliland in 1960. Following a civil war and the collapse of the central government of Somalia, the people in the former British Somaliland Protectorate declared independence in 1991 and formed the Republic of Somaliland. Although Somaliland has not received international recognition, it has managed to secure peace and stability for almost 30 years.^2^ The population was estimated to be 3.8 million in 2017, with 45% living in rural areas. Farming livestock is the backbone of the Somaliland economy.

The health of the Somaliland population is poor. Life expectancy is estimated at 53 and 56 years for males and females, respectively. One in seven children dies before their fifth birthday, and 1 in 18 women has a lifetime risk of death during pregnancy.^3^

In 2009 the government of Somaliland adopted an Essential Package of Health Services (EPHS) as the framework for healthcare delivery by the public sector. The EPHS provides service at four levels of care. The primary health unit and the health centre provide primary healthcare and are staffed by non-physician providers. The primary hospital (referral health centre, district hospital) is meant to be staffed by general practice doctors, and the regional hospital is organised around specialist doctors. Currently about half of the population has access to the EPHS.^2^

Somaliland’s first Faculty of Medicine was established at Amoud University in 2000. There are now four recognised medical schools in the country. In 2012 Amoud University initiated a 3-year master’s degree programme in Family Medicine. This was the first specialty training programme in the country. Its purpose is to prepare generalist doctors to provide patient care at primary hospitals and to work alongside primary care practitioners serving the catchment population of the hospital. Family Medicine training was an initiative of the university, not the government. At present, there are no other Family Medicine programmes in the country.

In Somaliland there is no obligation to serve the government following graduation from medical school. Once they receive their degree, doctors are free to practise or to seek specialisation. Following specialisation there is again no obligation, and specialists are free to seek employment wherever they wish.

## The supply of family physicians

In May 2021, a cross-sectional survey was carried out amongst the 29 graduates of the Amoud Family Medicine training programme (Table 1). Five graduates (17.2%) were excluded from further analysis, because they had emigrated from the country. The mean age of the other 24 respondents was 33 years, and there was an equal split of men and women. Most graduates worked in urban areas, were employed in a primary hospital and were teaching at a university. The mean number of employers was 2.1. The majority have also been pulled into leadership and management positions (62.5%).

**TABLE 1:** Characteristics of the first family physicians in Somaliland.

Variable	*n*	%
Male	12	50.0
Female	12	50.0
Urban practice	23	95.8
Rural practice	1	4.2
Employed in public hospital only	8	33.8
Employed in private hospital only	6	25.0
Employed in both public and private hospitals	10	41.7
Employed in primary hospital	20	83.3
Doing C-sections routinely	15	62.5
Teaching at a university	20	83.3
In a management position	15	62.5

*n* = 24.

## Discussion

The fact that graduates working in Somaliland have an average of 2.1 employers confirms that there is no shortage of work opportunities for family physicians. Many graduates (41.7%) are working in both the public and private sectors. Because a main goal of the Ministry of Health Development is ‘to contract the private sector to provide public health services at affordable prices’,^3^ this willingness to engage both sectors could represent an opportunity to strengthen the health system through public–private partnerships.

Half the Family Physician graduates employed in Somaliland are women. This aligns with a priority objective of the Somaliland Health Sector Strategic Plan (HSSP), ‘to increase access to and utilization of cost-effective, quality and gender-sensitive health services especially for women, children, and other vulnerable groups by 2021’.^3^ It also aligns with Somaliland’s Education Sector Strategic Plan objective ‘to expand and increase access and equity in higher education’.^4^

Somaliland’s family physicians follow the common pattern of doctors settling in urban areas (96% employed in urban settings), which leads to weaknesses in health delivery in rural areas. To encourage a more even distribution of family physicians throughout the country, the training programme could intentionally recruit candidates from rural areas, registrars could be offered experiences in rural practice during training, and the public and private sectors could increase incentives for rural practice. Alternatively, a second Family Medicine training programme could be established in a rural area of the country.

The purpose of Family Medicine training is to strengthen the Somaliland health system by preparing doctors to work effectively at the level of the primary hospital. Of the 24 graduates employed in Somaliland, 20 (83.0%) are working in primary hospitals. These results suggest that the training programme is achieving its purpose. The fact that 15 graduates (62.5%) are routinely doing C-sections contributes to achieving the highest priority objective of the HSSP: to reduce maternal, neonatal and child mortalities.^3^

Most graduates have academic positions in public and private universities (83.0%). This aligns with the Education Sector Strategic Plan objective ‘to build institutional and human capacity at all levels of the education ministry to facilitate implementation of education reforms.’^4^ Universities hire graduates to teach medical students and Family Medicine registrars. Family physicians multiply the impact of their own training by teaching others. The Family Medicine curriculum anticipates these opportunities and includes instruction on how to teach.

Fifteen graduates (62.5%) are employed in leadership positions, including the following: one dean and one associate dean of a medical school, four hospital directors, three medical directors, and the director of the Family Medicine training programme. The number three priority of the HSSP is ‘to strengthen the leadership, governance, institutional and management capacity of the health sector to deliver efficient and effective health programmes and services’.^3^ These graduates are well placed to achieve that priority. Whilst the focus of the Family Medicine programme is to train clinicians, the Family Medicine faculty should recognise this leadership gap in the country and strengthen its curriculum in leadership and management.

Five out of 29 graduates are not employed in Somaliland because of emigration. Unfortunately, these five graduates are not part of the Somaliland health system. The loss of 17.0% of the graduates to emigration should lead the Family Medicine faculty to explore ways to increase retention.

## Conclusion

Family physicians in Somaliland provide clinical skills and leadership in public and private sectors to strengthen the health system, especially at the level of the primary hospital in urban areas. Most family physicians in Somaliland also contribute to higher education by teaching. Somaliland could strengthen its health system further by scaling up Family Medicine training.
